# Synergism and Combinatorial Coding for Binary Odor Mixture Perception in *Drosophila*

**DOI:** 10.1523/ENEURO.0056-14.2016

**Published:** 2016-08-23

**Authors:** Srikanya Kundu, Anindya Ganguly, Tuhin Subhra Chakraborty, Arun Kumar, Obaid Siddiqi

**Affiliations:** National Centre for Biological Sciences, Tata Institute of Fundamental Research, GKVK Campus, Bangalore 560065, India

**Keywords:** behavior, binary mixture, Drosophila melanogaster, neuronal coding, receptor specificity, synergism

## Abstract

Most odors in the natural environment are mixtures of several compounds. Olfactory receptors housed in the olfactory sensory neurons detect these odors and transmit the information to the brain, leading to decision-making. But whether the olfactory system detects the ingredients of a mixture separately or treats mixtures as different entities is not well understood. Using *Drosophila melanogaster* as a model system, we have demonstrated that fruit flies perceive binary odor mixtures in a manner that is heavily dependent on both the proportion and the degree of dilution of the components, suggesting a combinatorial coding at the peripheral level. This coding strategy appears to be receptor specific and is independent of interneuronal interactions.

## Visual Abstract

**Figure:**
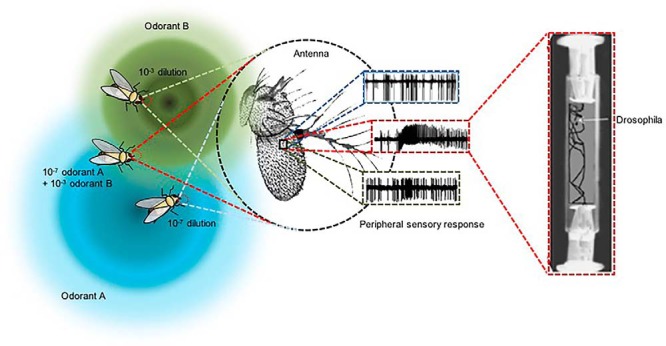

## Significance Statement

Insects rely on olfaction to successfully identify and distinguish between volatile chemical cues that are essential for reproduction and survival. Most naturally occurring olfactory signals are complex mixtures of many chemicals in varying compositions and proportions. In the present study, using *Drosophila melanogaster*, we have shown that the olfactory system can encode the information for binary odor mixtures by exhibiting a strong response toward specific combinatorial concentrations independent of the individual odor intensities. We further found that the ratio coding is receptor specific and is independent of ephaptic interactions. The particular combinations could be relevant in terms of the ecology of the fly. This study can also pave the way toward better understanding of the mechanism of host tracking by insect pests and vectors.

## Introduction

Most odors that animals encounter in their natural environment are complex mixtures of many chemicals. Yet, ironically, most studies in olfaction have been directed toward sensing single odor, and different aspects of sensing odor mixtures have remained unexplored. Previous studies suggested that the identity of individual components is lost in the blend and, as a result, odor mixtures are perceived differently (Freitag et al., 1998; Syed and Guerin, 2004; Rebora et al., 2012, 2013; Faucher et al., 2013; Roussel et al., 2014; Schütze et al., 2014). The measured response to an odor mixture is often inconsistent with predictions based on measured responses to the individual components contained in the mixture (Deisig et al., 2006; Eschbach et al., 2011; Barth et al., 2014). For example, in rats, the magnitude of the response of the olfactory sensory neurons (OSNs) to odor mixtures is different from the simple summation of the individual components (Duchamp-Viret et al., 2003). Among the various types of interactions observed with odor mixture, the most frequent interaction is suppression, where one of the odors cancels the response to the other. However, OSNs are also capable of exhibiting synergism, a less well documented phenomenon, where the response of a binary mixture is greater than the simple summation of the individual components present (Deisig et al., 2006). Synergistic interactions among the components of an odor blend have been proposed to contribute to the attraction of some species of insects to their host plants (Visser, 1986). Empirical evidence for synergism between plant-derived volatiles and specific aromatic compounds or pheromones have been documented in the oriental fruit moth (Piñero and Dorn, 2007), and in the males of the codling moth (Yang et al., 2004) and corn earthworm (Ochieng et al., 2002). In this study, we investigate whether synergized responses are encoded in the OSNs, using *in vivo* extracellular single-unit recording and behavioral studies in the relatively simple and well defined *Drosophila melanogaster* olfactory system.

Odors are first recognized by a large repertoire of olfactory receptors (ORs), each of which is expressed in a specific class of OSNs (Gao et al., 2000; Vosshall et al., 2000). OSNs expressing a specific odorant/class of odorant receptor project their axons to the antennal lobe, where they synapse onto the dendrites of the corresponding classes of projection neurons (Stocker et al., 1990; Jefferis et al., 2001). Both insect and mammalian olfactory systems share many of these organization principles, indicating a common solution to odor mixture representation in the higher brain centers (Davis, 2004; Silbering and Galizia, 2007; Su et al., 2009; Chou et al., 2010; Wilson, 2013). In honeybees, it has been suggested that the peripheral olfactory neuronal layout hardly contributes to the mixture perception (Deisig et al., 2006, 2010; Huang et al., 2009). However, accumulating evidence suggests that the odor-evoked responses at the OSN level are also important, as they transmit key information about odor quality to the brain (Den Otter, 1977; Nikonov et al., 2002; Su et al., 2012). Few recent studies have reported that odor-guided behavior can be correlated to the activity of sensory neurons (Semmelhack and Wang, 2009; Knaden et al., 2012). Hence, in order to understand the behavior of a fly toward odor mixtures, a detailed understanding of the response patterns in the OSNs and the underlying mechanisms is crucial.

We focused our study on type I and type II sensilla basiconica (ab1 and ab2, respectively; Stensmyr et al., 2003) of *D. melanogaster* housing four and two OSNs, respectively. They mostly detect food odors including esters, alcohols, and aldehydes (de Bruyne et al., 2001; Chakraborty et al., 2009). We tested a set of odorants (and their binary mixtures) representing the ligands for these two sensilla types as reported by previous studies (de Bruyne et al., 2001; Hallem and Carlson, 2004). We observed a synergistic effect in response to the binary mixtures in the OSNs. We further noticed that these sensory neural responses to binary odor mixtures were also reflected in the behavior of the fly (i.e., synergistic interaction between two odorants significantly increases the attraction of the fly toward their binary mixture). Although the results presented here cover only a small part of an enormous number of chemical stimuli that a fruit fly encounters in the natural environment, this finding suggests that the enhanced behavioral response to the odor mixture is at least in part due to the increased firing of OSNs.

## Materials and Methods

### Flies

Two- to three-day-old adult female *D. melanogaster* [Canton-S Benzer (CsBz)] flies were used for the experiments. Flies were reared on standard cornmeal medium at 24°C and 40–50% relative humidity under laboratory photoperiod regime (∼14/10 h light/dark cycle). These flies were used throughout all of the experiments, unless specified otherwise.

For the UAS-reaper (rpr) experiments, ∼7-d-old flies were used for recording. The flies were exposed to 29°C for 4–5 d to enable a higher expression of reaper so as to ensure cell death.

### Preparation of flies

Female flies were anesthetized by cold shock for 20 s on ice and mounted in a 1.6-mm-diameter glass capillary (Harvard Apparatus). The protruding head was immobilized with a low-melting-point myristic acid (Himedia). Extra care was taken while mounting the fly in order to protect the sensilla from heating. The proboscis was also fixed sideways so as to prevent any movement

### Odor preparation and odor delivery

A custom-made five-port olfactometer made up of a three-way open solenoid valve (Model LFAA1200118H, Lee Company) was used to deliver the odor pulses. Twenty milliliter scintillation vials were used as odor reservoirs, and 2 ml dilutions of the desired odor (Sigma-Aldrich) were placed in each vial. All of the odorants were prepared by serial dilution in liquid paraffin oil (SD Fine Chemical). Odor mixtures were premixed just before delivery. The purity of the diluted chemicals was further checked by a Gas Chromatography Mass Selective Detector (Model 6890 and Model 5973, Hewlett Packard). A small volume of constant air was injected through the inlet reservoir into the chemical scintillation vials, which eventually forced the odor-saturated air to come out through the outlet of the vial. Odor pulses were delivered through a 2.00 mm glass tube directed toward the fly antennae at a rate of 9 ml/min. The duration of each odor stimulation was 500 ms, and was controlled electronically and back fed to LabVIEW version 7.1 (National Instruments) acquisition software. The time taken by the odor to travel from the outlet to the antennae, and the delay between valve opening and the onset of the response were standardized before the experiments started and were maintained throughout the experiments.

### *In vivo* single-unit extracellular recording

**Figure 1.**
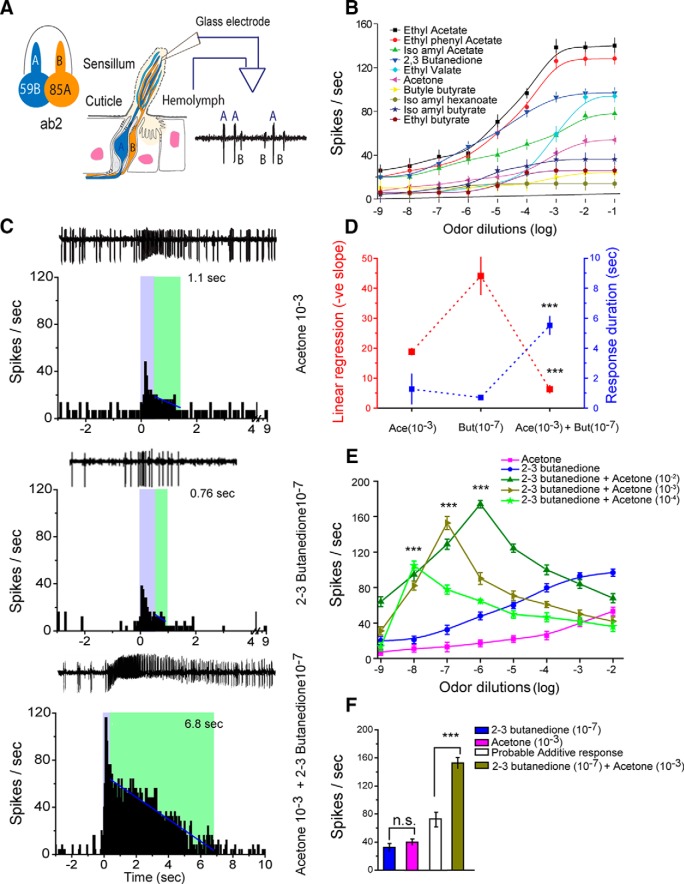
*In vivo* extracellular single-unit recording from ab2. ***A***, Schematic diagram of the recording set up. Trace shows two olfactory sensory neurons expressing Or59b and Or85a, labeled as “A” and “B,” distinguished by larger- and smaller-amplitude spikes, respectively. ***B***, Dose–response curves of individual ligands. ***C***, Representative traces and corresponding PSTHs in response to 500 ms (marked as blue) stimulation by acetone (10^−3^), 2,3-butanedione (10^−7^), and their binary odor mixture. The binary mixture of acetone (10^−3^) and 2,3-butanedione (10^−7^) had an enhanced response magnitude (i.e., firing frequency as well as prolonged response, marked as green, sustained for longer time). ***D***, The total duration of the odor response for the binary mixture is 6.8 s and was sustained longer than its individual component responses, 1.1 and 0.76 s, respectively, for acetone and 2,3-butanedione. On the other hand the linear regression of response decay was significantly lower for binary mixture than it's individual component alone. ***E***, The dose–response curve of the binary mixture did not follow the typical sigmoidal pattern. For each dilution set of acetone (10^−4^/10^−3^/10^−2^), the odor mixture-evoked firing rate steeply increases up to a certain dilution of 2,3-butanedione and formed a sharp peak at 10^−8^/10^−7^/10^−6^, respectively. ***F***, Bar graph shows that the observed response of the binary mixture of acetone and 2,3-butanedione was significantly higher than the expected additive response. Responses were compared in paired-sample *t* test. Error bars represent the SEM; ***p = 0.001. *N* = 30.

Large sensilla basiconica on the third antennal segment were identified by their position using a standard map of the antennal surface (de Bruyne et al., 2001). Glass microelectrodes of 5–10 MΩ were prepared by pulling glass capillaries (catalog #121411, Kimble Chase) on a vertical electrode puller (Narishige Scientific Instrument Laboratory) and filling them with 0.8% NaCl solution. Chlorided silver wire was used for making electrical connection with the electrode. The ground electrode was placed inside the third antennal segment, and the recording electrode was placed on the base of a type I/type II basiconic sensillum and connected to a high-impedance unit gain pre-amplifier (Electro 705, WPI), as shown in Figure 1A. Signals were amplified 1000× (Model 750, World Precision Instruments), viewed on a two-channel oscilloscope (Model 2216, Tektronix) that was linked to an audio amplifier, and fed into a computer via a 16 bit analog-to-digital converter (NI-DAQ BNC-2110, National Instruments) to be analyzed off-line with LabVIEW version 7.1 software (National Instruments) or Clampfit.

### Single-fly behavioral assay

Two- to three-day-old female flies were separated in vials containing a moist bed of tissue paper. For behavioral experiments, five flies were tested per set in five separate glass tubes. At least five sets were performed for each experiment. Each glass tube was closed on both ends using an apparatus made up of a microfuge tube (Tarson) with the tip cut off to a diameter of 4 mm and replaced with a micropipette tip so that odor could pass from the microfuge tube into the glass tube. Clean plastic mesh with a diameter of 11 mm and a pore size of ∼1 mm was attached to the mouth of the apparatus to prevent the flies from entering the mouth of the trap. The odor was applied on a filter paper disc placed on the cap of the microfuge tube at one of the arms (odor arm), and paraffin oil was applied on the other arm (control arm). An overhead camera (Monochrome Video Camera, Watec) was used to record the movement of the flies for 2 min at 25 frames/s. Fly-tracking analysis was performed using a custom-written program in MATLAB version 6.5 (R2007b; MathWorks) that tracks the path of the each individual fly in each tube. A fly covers a mean distance of 623 ± 48 mm in 2 min in the absence of odor. The total time spent by the fly at the odor arm was calculated. The response index (RI) was calculated using the following formula: (time spent in odor arm − background)/total time of the analysis (2 min).

### Data analysis

**Figure 2.**
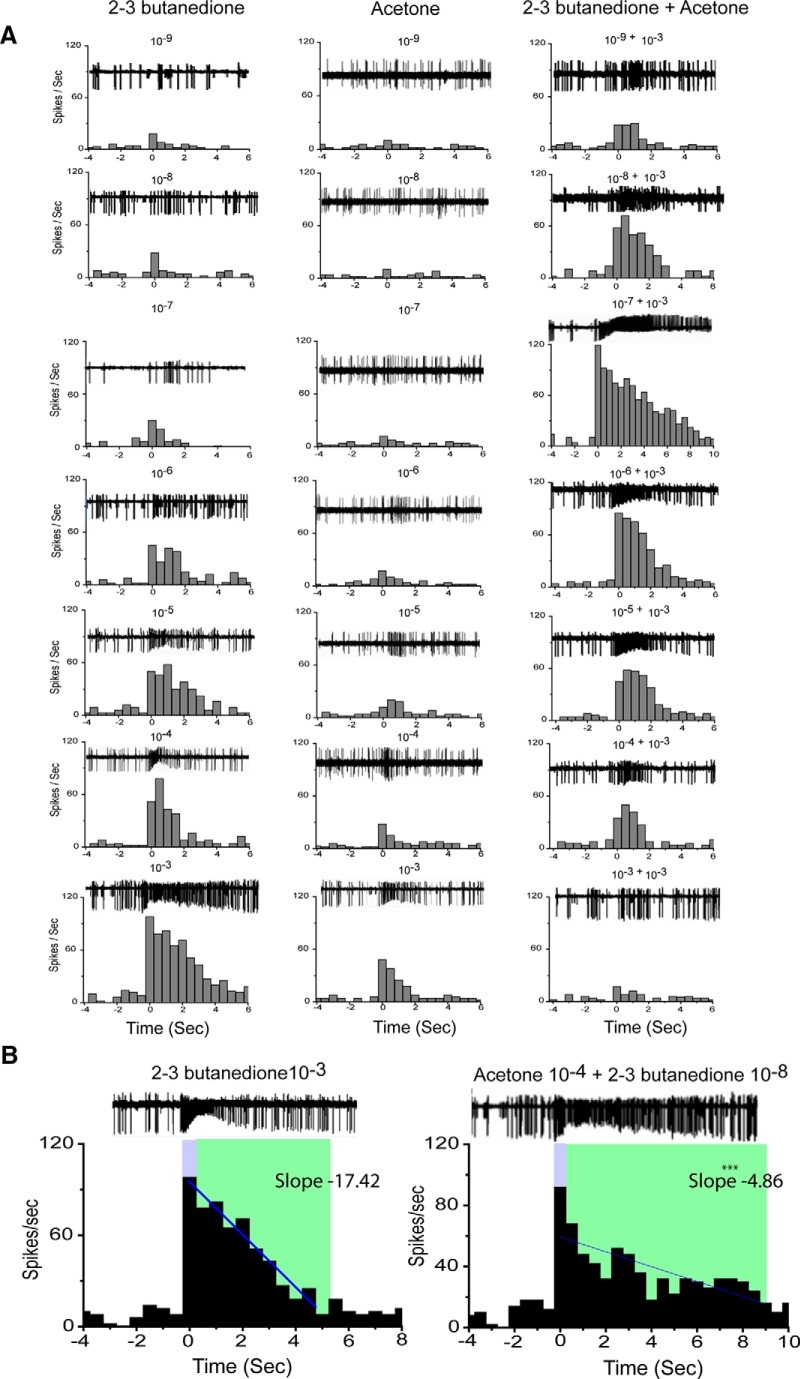
Extracellular recording and their corresponding PSTH in response to serial dilution of acetone, 2,3-butanedione, and their binary mixture from ab2. ***A***, Representative traces of Or59b-expressing neuron responses toward serial dilutions from 10^−3^ to 10^−9^ of acetone, 2,3-butanedione, and the binary mixture of serial dilution of 2,3-butanedione with 10^−3^ dilution of acetone. Both acetone and 2,3-butanedione alone showed concentration-dependent increased firing frequencies. The odor-evoked responses of the binary mixture were not linear. The maximum response magnitude observed at a 10^−7^ dilution of 2,3-butanedione when presented with acetone (10^−3^). ***B***, The linear regression of the odor-evoked response of 2,3-butanedione (10^−3^) alone is significantly (*p* > 0.001) faster than that of the binary odor mixture of acetone (10^−4^) and 2,3 butanedione(10^−8^). The duration of odor stimulation was 500 ms, marked in blue, and the total response duration was marked in green. The solid blue line indicates the linear regression.

All the graphs and the statistical analysis mentioned in the experiments were performed using Origin version 9.1 software. For Figure 2, spike sorting was carried out using a Plexon off-line sorter, and frequency histograms were plotted in Neuroexplorer with 500 ms bin width. We calculated the average basal firing rate prior to the stimulation for 2 s. Starting at the zero point of the stimulation, we determined the point where the evoked response came down to the same basal firing rate, and was marked as green and considered as the response duration. The linear regression (blue solid line) plots of the response duration were drawn using Origin version 9.1 software. The slopes of the decay plot for 2,3-butanedione (10^−3^) and the binary mixture of 2,3-butanedione (10^−8^) and acetone (10^−4^) were then compared using a paired *t* test for significant analysis.

## Results

Flies extract biologically relevant information from the environment via chemical signals detected by a large array of ORs present in the sensilla of each antenna. In the present study, we focused our attention on Or42b-expressing neuron and Or59b-expressing neuron, housed in ab1 and ab2, respectively. We tested all the odorants to which Or59b-expressing neuron (Fig. 1B) are sensitive, as identified in previous studies (de Bruyne et al., 2001).

**Figure 3.**
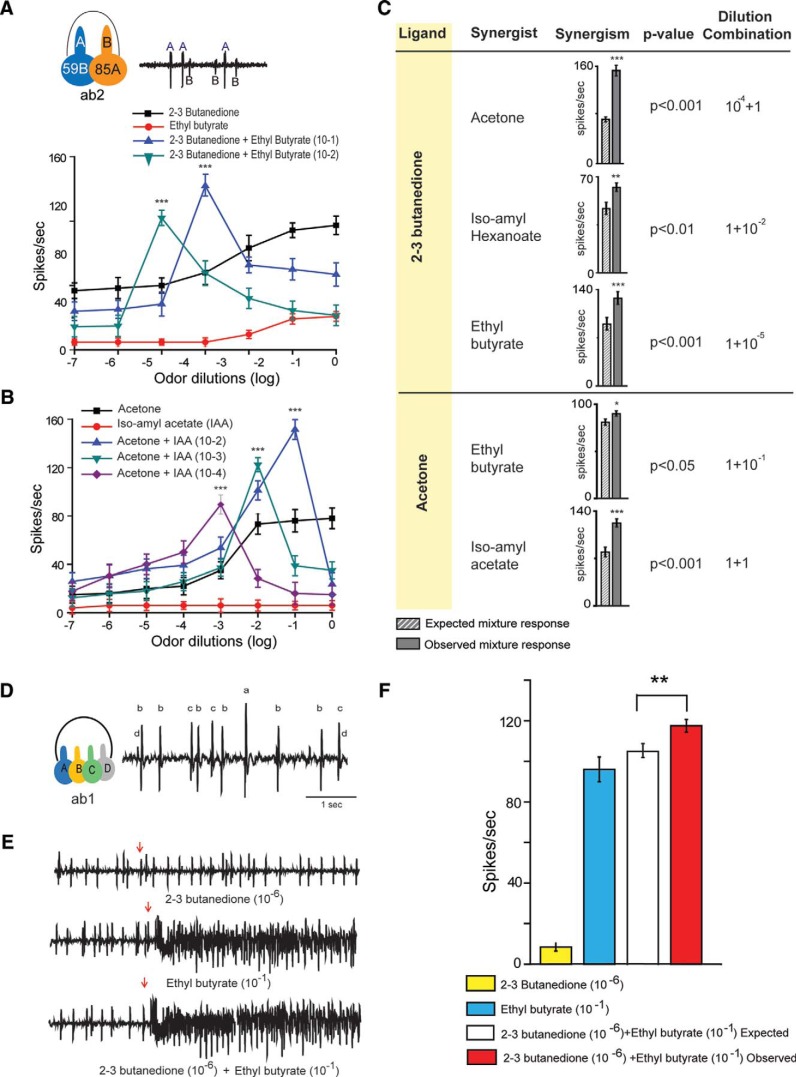
Synergistic responses in ab2A and ab1B. ***A***, ***B***, Extracellular recording from Or59b-expressing neurons showed synergistic interaction between 2,3-butanedione with ethyl butyrate (***A***) and acetone with iso-amyl acetate (***B***). Iso-amyl acetate did not evoke any response to Or59b-expressing neurons, but in a binary mixture with acetone, synergism was observed (***B***). The peak responses of these two binary odor combinations observed at 1:10^−5^ and 1:1 dilution combinations of 2,3-butanedione and ethyl butyrate, and acetone and iso amyl acetate, respectively (***A***, ***B***). ***C***, The list of binary mixture combinations that exhibit synergism in ab2. A paired *t* test reveals that the observed synergistic peaks were always significantly greater than the expected additive response of their corresponding mixture components. *N* = 30. ***D***, Trace showing the spontaneous firing of a typical ab1 sensillum. Spikes obtained from different neuronal types within the sensillum have been marked accordingly. ***E***, Representative traces for the responses elicited by 2,3-butanedione (10^−8^), ethyl butyrate (10^−1^), and the binary mixture of the aforementioned chemicals recorded from ab1 sensillum. ***F***, Bar graphs showing the quantification of average electrophysiological responses elicited from ab1B neuron by 2,3-butanedione (10^−8^), ethyl butyrate (10^−1^), and a mixture of the aforementioned chemicals. *N* = 12. Error bars represent the SEM. *p = 0.5, **p = 0.01, ***p = 0.001.

Extracellular single-unit recordings were acquired from ab1 and ab2 sensilla with glass electrodes. For the ab2 sensillum, the larger amplitude spike marked “A” and the smaller amplitude spike marked “B” (Fig. 1A) represented responses obtained from OR neurons with Or59b and Or85a receptors, respectively (Pellegrino et al., 2010). The firing frequency of ‘A’ spikes was measured throughout the experiment. For ab1 sensillum, all four (A, B, C, and D) amplitude spikes (Fig. 3D) were recorded, but only spike B was used for experimental consideration. Firing rates were obtained from OSNs from 10 flies (3 OSNs from each fly per odorant) using *in vivo* extracellular recording in the presence of varying odorant concentrations. Appropriate controlled conditions for the olfactometer were maintained throughout the experiment (see Materials and Methods). A dose–response curve for each odorant was then generated. For complete quantitative analysis of the dose–response curve, two criteria were used. First, for all 10 odorants, data points encompassing the entire dynamic range were included, up to the dilution close to the saturation vapor (dilutions range, 10^−9^ to 10^−1^). Second, at least three trials for each dilution set were included. We carefully measured the responses of the odorants across different concentrations. Our results, shown in Figure 1B, suggested that all 10 odorants exhibited a sigmoidal relationship between the concentration and the firing rate, as already described. The 10 tested odorants were grouped into three classes based on the firing frequency of the OSN response, an indicator of the affinity of Or59b receptors toward the odorant. Class I odorants evoked the highest firing rates (>100 spikes/s) at their dynamic range, class II odorants evoked a moderate response (>50–100 spikes/s), and class III odorants evoked the lowest firing rate (< 50 spikes/s). While selecting odorants for preparing binary mixtures, no two odorants from class I were combined to avoid reaching firing frequency saturation at very low concentrations.

For ab1 sensillum, four neurons can be easily identified from the spontaneous firing (Fig. 3D). In our study, it has been noted that the spontaneous firing of ab1 sensillum is much higher than that of the ab2 sensillum. We focused on the Or42b receptor, which is housed in the ab1B sensory neuron. The responses of 2,3-butanedione and 3-hydroxyethyl butyrate in ab1B were consistent with those from earlier studies (de Bruyne et al., 2001).

### Single odor and binary odor mixture responses in ab1 and ab2

Responses to each set of two individual odorants and their binary mixture were tested. Single-unit responses for acetone and 2,3-butanedione were measured from ab2, both individually and mixed together (Figs. 1C–E, 2). Subthreshold levels of acetone (10^−3^) and 2,3-butanedione (10^−7^), when mixed together, evoked a stronger and more sustained response than the individual components alone. The green area in Figure 1C indicates that the response extended beyond the length of the odor stimulus (i.e., 500 ms; the blue box). Typical excitatory responses returned to the spontaneous firing level within 1.1 and 0.76 s after the end of the odor stimulus, as illustrated by the responses elicited by acetone (10^−3^) and 2,3-butanedione (10^−7^), respectively, when presented alone (Fig. 1C,D). In contrast, a binary mixture of 2,3-butanedione (10^−7^) and acetone (10^−3^) elicited a more sustained response of 6.8 s. The linear regression fittings (Fig. 1C, blue solid line) of the response durations of the two individual odorants and their binary combination suggested that the binary mixture of these two odorants elicited a supersustained response. To understand the synergistic effect of the odor mixture, we mixed different concentrations of 2,3-butanedione and acetone, and tested them on Or59b-expressing neurons. Each mixture set contained a fixed dilution of acetone (10^−4^, 10^−3^, or 10^−2^) with serial dilutions of 2,3-butanedione (10^−9^ to 10^−2^; Fig. 1E). However, the dose–response curves for the binary mixtures of 2,3-butanedione and acetone exhibited the following dramatic effect: each mixture set showed a sharp increase in firing frequency at lower concentrations of 2,3-butanedione (Fig. 1E) followed by a sharp peak at a certain dilution combination of 2,3-butanedione and acetone, which attenuated at higher concentrations of 2,3-butanedione. Each peak was significantly (one-way ANOVA, *p* = 0.001) greater than the peak witnessed in response to 2,3-butanedione alone of that corresponding dilution, which suggested synergism at that dilution. When 2,3-butanedione was mixed with a 10^−3^ dilution of acetone, the response of the binary mixture peak/synergism appeared at a 10^−7^ dilution of 2,3-butanedione, which was significantly (paired *t* test, *p* = 0.001) greater than the combined response of 2,3-butanedione at 10^−7^ and 10^−3^ dilutions of acetone, which are shown in the bar plot in Figure 1F. The maximum frequency was documented only at particular proportions. The highest response synergism for 10^−8^ and 10^−6^ dilutions was found for the binary mixtures of 2,3-butanedione when presented with 10^−4^ and 10^−2^ dilutions of acetone, respectively (Fig. 1E). Responses to the individual odorants and to their mixture were obtained from at least 30 neurons. Notably, comparing the combinations of varying dilutions of acetone with a fixed concentration of 2,3-butanedione, it was found that the peak responses to the binary mixtures were obtained at different dilutions of acetone, indicating that 2,3-butanedione showed synergism with a specific dilution combination of acetone. The particular dilution of 2,3-butanedione in the binary mixture combination that exhibits synergism was always found to be four times lower than the dilution of acetone. The firing frequency at a higher dilution (10^−9^) of 2,3-butanedione presented with acetone (10^−4^), however, appeared to be slightly lower than the firing response of 2,3-butanedione alone at that specific dilution shown in Figure 1E. But this difference is statistically (one-way ANOVA) insignificant. The representative traces of these two odorants and their combinatorial responses over the serial dilutions have been presented in Figure 2. The peristimulus histogram indicates that the responses (firing frequency) to both of the individual odorants gradually increased with their increasing concentration (Fig. 2, first and second columns). In contrast, the firing frequency for the binary mixture of 2,3-butanedione and acetone (10^−3^) was remarkably different. The peristimulus time histogram (PSTH) indicates the maximum firing frequency and longest sustainable response at the binary combination of 2,3-butanedione (10^−7^) and acetone (10^−3^).

Figure 2B showed the PSTH of 2,3-butanedione (10^−3^) when presented alone, and the binary mixture of 2,3-butanedione of 10^−8^ dilution and acetone of 10^−4^ dilution. Although the first 500 ms of the odor-evoked responses were not different (92 and 98 Hz, respectively), the response profiles of these two responses were notably different. The odor-evoked response duration for 2,3-butanedione (10^−3^) alone was 4.5 s, which was significantly (paired *t* test, *p* > 0.001; Fig. 2B, green area) shorter than the duration of the evoked response of the above-mentioned odor mixture (9 s). The time when the firing frequency over a 500 ms period reached the basal firing rate was recorded as the response time. More importantly, the linear regression/decay response slope (Fig. 2B, solid blue line) of the mixture of 2,3-butanedione (10^−8^) and acetone (10^−4^) was −4.86, which was significantly (paired *t* test, *p* > 0.001) slower than the response decay slope of −17.42 for 2,3-butanedione (10^−3^) alone. Together, our observations strongly suggest that the prolonged response of the binary mixture was not a result of vapor pressure differences in the individual components. The response profiles presented in Figure 2 clearly showed that the evoked responses of the binary mixtures were not due to sequential responses of the corresponding mixture components at any point.

Synergism was not found to be restricted to the binary mixtures of 2,3-butanedione and acetone for ab2. We could observe synergistic responses being elicited by binary mixtures of 2,3-butanedione and ethyl butyrate (Fig. 3A), and also by acetone in combination with isoamyl acetate (Fig. 3B). For the binary mixtures of 2,3-butanedione and ethyl butyrate, the synergism always appeared when the dilution of 2,3-butanedione was five times less than the dilution of ethyl butyrate. For the binary combinations of acetone and iso-amyl acetate, synergism was observed when the dilution of acetone was two times less than that of isoamyl acetate (Fig. 3B). Interestingly, iso-amyl acetate fails to activate ab2 neurons (de Bruyne et al., 2001) but shows a synergistic response when presented with acetone. We have also found several other binary combinations (Fig. 3C) for which ab2 sensilla basiconica exhibited synergism.

In order to investigate whether the above-mentioned synergism is restricted to Or59b-expressing neurons or is a general phenomenon across all the ORs, we decided to study Or42b which are expressed in the ab1B neuron. We chose 2,3-butanedione and ethyl butyrate, because the Or42b-expressing neuron is known to detect both (de Bruyne et al. 2001). We found that their mixtures at certain combinations could evoke synergistic responses (Fig. 3E,F). We tested binary mixtures of a 10^−6^ dilution of 2,3-butanedione and a 10^−1^ dilution of ethyl butyrate (Fig. 3E, as they were known to be synergistic in case of Or59b-expressing neurons, Fig. 3C) as well as the individual dilutions of each of the components. The mixture was found to elicit a response higher than the summation of responses (paired *t* test, *p =* 0.01) evoked by the binary mixture of 10^−6^ dilution of 2,3-butanedione and 10^−1^ dilution of ethyl butyrate when tested individually (Fig. 3F). These observations tempt us to hypothesize that the synergism is probably a common coding mechanism used by the OSNs across the olfactory system of the fly in order to perceive complex odor mixtures that are present in the environment. Quite interestingly, the synergistic effect observed for ab1B (Or42b-expressing neurons) neuron was not as dramatic as that observed for ab2A (Or59b-expressing neurons).

### OSNs synergistic response conveys to the behavior of the fly

**Figure 4.**
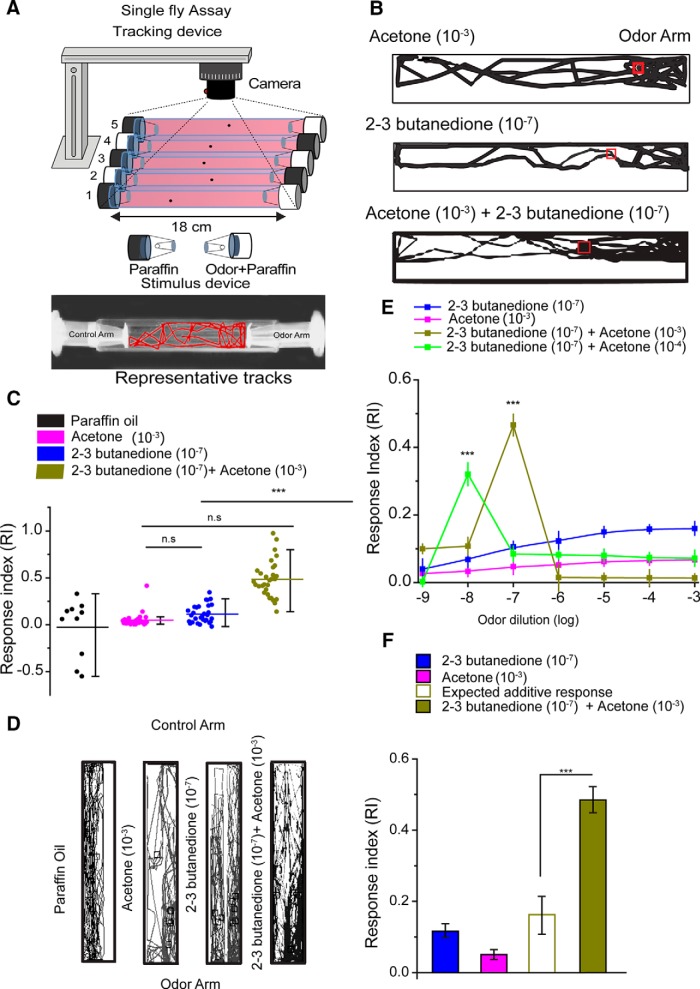
The binary mixture of acetone and 2,3-butanedione evokes a synergistic response in the behavior of a fly. ***A***, The schematic representation of the single-fly behavioral setup. ***B***, Representative traces from a single-fly response to acetone (10^−3^), 2,3-butanedione (10^−7^), and the binary mixture of 2,3-butanedione (10^−7^) and acetone (10^−3^). ***C***, The response of the fly toward the binary mixture of acetone (10^−3^) and 2,3-butanedione (10^−7^) was significantly higher (one-way ANOVA, *R*^2^ = 0.57) than the individual odorants. ***D***, The population fly tracks of 30 flies indicate that flies spent more time at the odor arm of the binary mixture than their corresponding mixture components. ***E***, The behavior of the fly over serial dilutions of 2,3-butanedione with acetone (10^−4^/10^−3^). At a specific dilution, combinations of the two odorants exhibited higher responses by the fly (*R*^2^ = 0.62 and *R*^2^ = 0.583). ***F***, The peak RI observed for the binary mixture of 2,3-butanedione (10^−7^) and acetone (10^−3^) is significantly greater in paired *t* test than the expected additive response of acetone (10^−3^) and 2,3-butanedione (10^−^7) alone. Error bars represent the SEM. ***p = 0.001.

In order to investigate whether the electrophysiological responses translate to the behavior of a fly, we conducted single-fly behavioral assays using the setup already described in Materials and Methods (Fig. 4A). Consistent with the electrophysiology, flies exhibited an enhanced response for the binary mixtures of 2,3-butanedione (10^−7^) and acetone (10^−3^) over the individual odors (Fig. 4B). The RI of all 30 flies and their response path tracks are shown in Figure 4, C and D, respectively. The flies exhibited enhanced attraction (ANOVA: *p* = 0.001, *R*^2^ = 0.72) to the combination of 2,3-butanedione (10^−7^) and acetone (10^−3^) compared with 2,3-butanedione (10^−7^) alone. To exclude any potential behavioral effect toward the solvent (paraffin oil), we tested the behavior of fly with paraffin oil on both the arms. Fly tracks in Figure 4D looked scattered/evenly distributed and showed no preference toward paraffin oil (Fig. 4C,D). We also measured the response of the fly to serial dilutions of 10^−9^ to 10^−3^ of 2,3-butanedione in two sets, each set containing a specific dilution of acetone (10^−4^ or 10^−3^; Fig. 4E). Surprisingly, consistent with the peripheral OSN responses in the single-fly behavioral RI curve in Figure 4E, the sharp peaks (ANOVA: *p* = 0.001, *R*^2^ = 0.42 and *R*^2^ = 0.66) were observed for both sets of acetone (10^−3^ and 10^−4^) and 2,3-butanedione mixture (10^−7^ and 10^−8^) combinations. So, the enhanced response toward the binary mixture was observed not only at the OSN level, but the fly also exhibited increased attraction toward the binary mixture too, which was significantly higher (paired *t* test, *p* = 0.001) than the additive response of the two individual odorants, 2,3-butanedione (10^−7^) and acetone (10^−3^) seen in Figure 4F.

More significantly, the corresponding combinatorial dilutions of 2,3-butanedione and acetone at which the highest response peaks were obtained in single-fly behavior (Fig. 4F) were exactly the same as those of the sensory neuronal level responses toward the same combinatorial binary mixture. Enhanced attraction was obtained at 10^−8^ and 10^−7^ dilutions of 2,3-butanedione when presented with 10^−4^ and 10^−3^ dilutions of acetone, respectively. Enhanced attraction toward 2,3-butanedione was found with a specific dilution combination of acetone. The dilution of 2,3-butanedione required to exhibit synergism both at the neuronal level as well as at the behavioral level was four times lower than the dilution of acetone in their binary combination.

### Synergism is receptor specific

**Figure 5.**
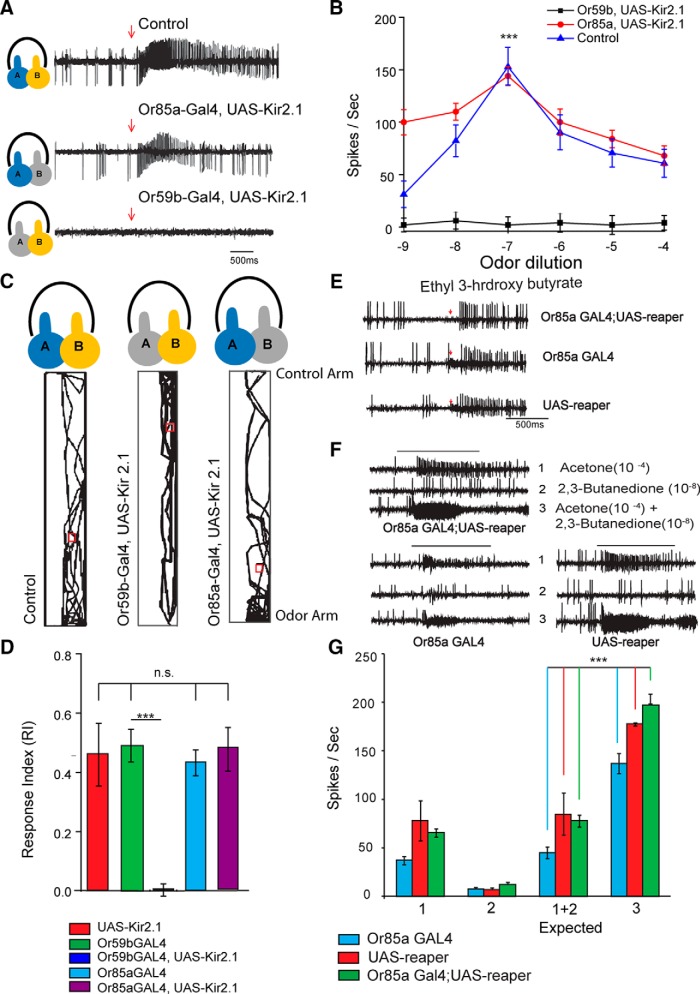
The synergism response toward a binary mixture is specific to the receptor. ***A***, ***B***, Representative traces and dose–response curves show the response profile toward the binary mixture of acetone (10^−3^) and 2,3-butanedione (10^−7^). Both the control and flies where Kir 2.1 was expressed in Or85a-expressing neurons exhibited a synergistic response to the binary mixture. ***C***, ***D***, Expressing Kir 2.1 in Or59b-expressing neurons abolished synergism (*N* = 30). ***D***, Flies with silenced Or59b-expressing neurons showed less of a response toward the binary mixture (ANOVA, *R*^2^ = 0.725). Gal4 and UAS-Kir2.1 controls maintained their synergistic phenomenon. ***E***, Representative traces for the responses elicited by ethyl 3-hydroxy butyrate from the ab2 sensilla from flies of three different genotypes compared with Or85aGal4,UAS-rpr and the UAS and Gal4 controls (*N* = 20). The larger spikes are from ab2A, whereas the smaller spikes are from ab2B. In Or85aGal4,UAS rpr, the ab2B spikes are absent, confirming cell death owing to reaper expression. The Gal4 and UAS control lines show a normal increase in ab2B spikes in response (indicated by arrows) to ethyl 3-hydroxy butyrate, confirming that there were not any background effects. ***F***, Representative traces obtained from ab2 sensillum of flies of two different genotypes, Or85aGal4,UAS-rpr and the UAS control, following stimulation by 2,3-butanedione (10^−8^), acetone (10^−4^), and a mixture of these two odors. ***G***, Bar graphs showing the quantification of average electrophysiological responses elicited from ab2A neurons of flies of three different genotypes (Or85aGal4,UAS-rpr, and the UAS and Gal4 controls) by 2,3 butanedione (10^−7^), acetone (10^−3^), a calculated summation of these two responses, and a response elicited by a mixture of these two odors. *N* = 6. Error bar represents the SEM. **p = 0.01, ***p = 0.001.

In order to determine whether the synergistic interaction observed at the sensory neuronal level was specific to the receptor, we used a Gal4-UAS system to drive either rpr or Kir2.1 in sensory neurons housed in the ab2 sensillum. The expression of reaper in ab2B sensory neurons led to cell death and abolished the spontaneous firing of Or85a-expressing neuron (Fig. 5E); whereas, the expression of an inward-rectifying Kir2.1 channel caused a voltage-dependent K^+^ ion channel opening, which shunted the membrane voltage toward the equilibrium potential of K^+^, thereby hyperpolarizing the neuronal membrane and making it refractory to synaptic activity (Baines et al., 2001). The crossed flies of reaper and Kir2.1 were tested electrophysiologically (Fig. 5 A,B,F,G). We also performed behavioral experiments with flies expressing Kir2.1. We found that flies failed to exhibit synergism toward the binary mixture of acetone (10^−3^) and 2,3-butanedione (10^−7^) when the excitability of the Or59b-expressing neuron was suppressed by Kir2.1 (Fig. 5A,B). In contrast, when Kir2.1 was expressed in Or85a-expressing neurons, synergism remained unaffected (Fig. 5A,B). Control flies in Figure 5B showed enhanced (ANOVA: *p* = 0.001, *R*^2^ = 0.6) firing toward the binary mixture of butanedione (10^−7^) and acetone (10^−3^). Similar results were obtained when reaper was expressed in ab2 sensillum. The expression of reaper in ab2B sensilla using a Or85a-Gal4 driver completely silenced that neuron because no ab2B spikes were seen when subjected to a puff of 10^−3^ ethyl 3-hydroxy butyrate, a natural ligand for Or85a-expressing neurons (Fig. 5E). However, normal Or85a-expressing neuron spikes were observed when the positive control fly lines (UAS and Gal4 lines) were puffed with 10^−3^ ethyl 3-hydroxy butyrate (Fig. 5E). Quite interestingly, the ab2A neuron was also seen to respond slightly to ethyl 3-hydroxy butyrate (Fig. 5E). The flies were then puffed with a 10^−3^ dilution of acetone and a 10^−7^ dilution of 2,3-butanedione, as well as their binary mixture. As expected, we saw synergistic responses in both the Or85a-Gal4-rpr flies as well as the UAS-rpr control flies (Fig. 5F-G). The responses of the Or85a- Gal4 line were slightly lower than those of the UAS line as well as the experimental reaper flies, but nonetheless they showed a synergistic response to the mixture. Together, these results strongly suggested that Or85a-expressing neurons did not physiologically contribute toward synergism. In both the Or85a-expressing neurons silenced and the control flies, we observed a lower response to 2,3-butanedione compared with that seen in Figure 1, which could probably be due to different age and growing conditions.

We further performed a single-fly behavioral assay with flies expressing Kir2.1 (Fig. 5C,D). As expected, flies exhibited significantly less attraction toward a binary mixture (paired *t* test, *p* = 0.001) when Or59b-expressing neurons were silenced (Fig. 5C,D). Flies spent almost no time in the odor zone when Kir 2.1 was expressed in ab2A sensory neurons. In contrast, control flies and flies with silenced ab2B sensory neurons (Fig. 5D) exhibited synergism. There were no significant differences between the RI values of control flies and those of ab2B silenced flies. These results strongly suggest that the Or59b receptors present in ab2 sensory neurons were solely responsible for the synergism witnessed.

## Discussion

In natural conditions, chemical stimuli are primarily complex mixtures of many chemicals. The mechanism by which the OSNs encode these complex mixtures is poorly understood. A recent study in *Drosophila* showed that the integration of information from odor mixtures begins in the OSNs in the form of the modulation of response dynamics and response magnitude (Turner and Ray, 2009; Su et al., 2012). Mixture interaction can therefore be observed prior to that in the higher brain centers at the sensory neuronal level in the periphery. It is tempting to say that the mixture interaction at the OSN level could shape the sensory input that the brain receives. Previous studies have shown that the most common interaction of odorants is suppression, where the addition of one odor attenuated the response magnitude for another odor (Steullet and Derby, 1997; Su et al., 2012). Here we have demonstrated another kind of interaction, where the presence of two odorants in a binary mixture significantly enhances both the response duration and magnitude, beyond that which would be expected based on an additive model of OSN response (Cometto-Muñiz et al., 1997). We refer to this phenomenon as synergism (Fig. 1). The dynamics of the odor coding for mixtures at the sensory neuronal level is highly selective. The presentation of mixtures of two odorants, such as acetone and 2,3-butanedione (at certain dilution combinations), elicited long-lasting evoked activities in ab1B and ab2A sensory neurons. The response extended beyond the length of the odor stimulus (Fig. 1C). The results we obtained in this study highlight the fact that the binary mixture response of sensory neurons cannot be predicted based on the individual component responses. The interaction appears complex even in the simplest case, where two odorants are mixed together. We speculate that the complexity would increase with the number of odorant molecules forming a mixture. We focused our study on two olfactory receptors, Or42b and Or59b, which are housed in the ab1B and ab2A OSNs. Both of these receptors have a broad profile with strong responses to many fruity odors, increasing the likelihood that the mixture interaction we recorded in this study could occur in the natural environment. In our study, we found that synergism also exists in other classes of OSNs, namely in ab1B (Or42b-expressing neurons) sensory neurons, where the synergism effect is not as severe as that in ab2A (Or59b-expressing neurons). It is possible that synergism is restricted to very selective cells responding in a context-dependent manner to import behavioral cues.

In this study, we used chemicals that are, by and large, attractive to the fruit fly. We observed that mixtures of these attractants (2,3-butanedione and acetone) were more attractive than each constituent alone. The cause behind the increased strength of the behavioral responses to the binary mixture probably lies in the temporal dynamics of the mixture responses. Our results support the observation made by Thoma et al. (2014) that compound valence is conserved in the binary mixtures. We went one step further and showed that the strongest synergism always occurs at a specific dilution combination within odorant pairs, independent of their individual absolute concentrations (Figs. 1–3). With changes in one odorant in the combinational pairs, the peak changes. Interestingly, the ratio coding observed is not uncommon; it appears to characterize pheromonal communication in moths (Baker et al., 1976; Lanier et al., 1980; Tòth et al., 1992) and beetles (Lanier et al., 1980), suggesting that such a strategy may be a general one for chemical sensing under natural conditions.

We also showed that the synergistic response was receptor specific. The expression of Kir2.1 in ab2A (Or59b-expressing neurons) sensory neurons abolished enhanced behavioral attraction to binary mixtures of 2,3-butanedione and acetone. We obtained similar results in extracellular single-unit recordings from the ab2 sensillum (Fig. 5A,E). However, we did not observe response reduction when the ab2B (Or85a-expressing neurons) sensory neuron was silenced (Fig. 5B). In order to strengthen our findings, we also expressed a cell death gene, rpr, in Or85a-expressing sensory neurons. Since there is a stereotyped pairing of OSNs in ab2 sensillum, the identity of the OSN that expresses rpr could be deduced from the identity of its surviving neighbor. We found that the surviving OSN in Or85a-Gal4, UAS-rpr flies had an odorant response that matched that of Or59b-expressing neurons (ab2A). Flies bearing Or85a-Gal4 and UAS-rpr showed no change in the response toward the binary mixture of 2,3-butanedione and acetone (Fig. 5F). Thus, from our results, we can strongly state that the ratio tuning we observed is not mediated by Or59b-expressing neuron–Or85a-expressing neuron interaction. Peripheral mechanisms such as ligand-induced receptor inhibition at the OSN level (de Bruyne et al., 1999; Hallem and Carlson, 2004; Su et al., 2012), ephaptic interaction within the sensillum (Su et al., 2012) or syntopic interaction (Münch et al., 2013) all require a system layout where receptors are inhibited by compounds, or positive and negative OSNs are colocalized within the same sensillum, to allow bilateral inhibition. We did not see such patterns in our study.

In general, the dose–response curve for an odor follows a sigmoidal shape. In our study, with synergism interaction, the response curve for the mixture did not look like that of a single compound, so evidently the brain processes the complex mixtures of multiple odorants differently from the pure constituents. The activity evoked from the Or42b-expressing neurons and the Or59b-expressing neuronal receptors studied here is only a small part of the whole ensemble of activity in the olfactory system. In the antennal lobe, the ensemble responses are further shaped by both excitatory and inhibitory lateral interactions conferred by a dense array of local interneurons (Chou et al., 2010). Nevertheless, we found that OSNs are the primary determinant for the behavior of the fly toward the perception of binary mixtures like 2,3-butanedione with acetone and many others. The exact mechanism by which the synergism occurs requires further investigation. It is possible that the ORs have multiple binding sites for different odorants, and binding at multiple sites leads to an amplified response. However, this also requires further study, such as expressing the Or59b and Or42b receptors in cell systems, which is beyond the scope of this study.

Together, our findings provide evidence that synergistic OSN responses are characteristic of the neurons and are not an outcome of bilateral interaction within the sensillum. Our study provides a better understanding of the strategies undertaken by the olfactory system of the fly in the presence of multiple odorants. We show that the insect olfactory system could encode information about chemicals at the olfactory receptor level as a separate entity, and recognizes them by segregating their component dilution combinations and not by their individual intensity. While information about the concentration is important for odor-guided behavior, it is often irrelevant for the purpose of odor recognition. In the current study, we showed that the olfactory system of the fly can deduce odorant identity independent of concentration when the two variables are intertwined at the level of receptor activity. The majority of odors consist of a combination of many chemical substances. The scent of banana, for example, was reported to contain 152 components (Jordán et al., 2001) and 26 substances in the headspace of fresh fruit. Whether the absolute concentration of the “main component” of banana is necessary, or whether the banana scent is only created by the specific dilution combination is an enigma. Here we show a case study where an effect similar to ratio coding happens at the receptor neuronal level.

## Synthesis

The decision was a result of the Reviewing Editor Darcy Kelley and the peer reviewers coming together and discussing their recommendations until a consensus was reached. A fact-based synthesis statement explaining their decision and outlining what is needed to prepare a revision is listed below. The following reviewer(s) agreed to reveal their identity: Xiaojing Gao.

Both reviewers and I appreciate the additional efforts that the authors have exerted re these interesting and provocative findings. In particular, their new presentation of spike data helps to dispel lingering "vapor pressure" doubts, and should be useful to readers interested in following up on the synergistic phenomenon.

Several additional issues remain, however, as discussed below.

One general issue is the question of under what circumstances, the reported synergism occurs, a concern triggered by the lack-of-synergism data in the previous draft and the new data presented in Fig. 5G. Might the reported synergism depend on factors such as the physiological state of the animal (for which they've seen experimental evidence)? Dependence on a fly's internal state would argue further against the vapor pressure caveat or any other trivial physicochemical alternative and suggest many interesting future directions that will hopefully shed light on the underlying mechanism.

Some specific additional issues are:

1) While the quantitative analysis of PSTH decay time is an interesting addition, the presentation is incomplete. Figure 2B is puzzling - why was this combination 2-3 butanedione at 10^-3 chosen and why was it compared it to a mixture of 2-3 butanedione at 10^-8 and Acetone at 10^-4? Are there published data in which a qualitative analysis of PSTHs demonstrate with certainty that the vapor pressures are not an issue? Have direct tests of vapor pressures (such as with a PID) in this experimental setup been employed?

2) Questions regarding the observation of inhibition at certain dilutions:

For example, in 3A and 3B, some mixtures result in lower firing rates than the individual components (i.e. 10^-3 2-3 butanedione + 10^-2 ethyl butyrate firing is ∼3x less than the firing elicited by 10^-3 ethyl butyrate alone). This result does not seem to jibe with the authors' response that "We have never seen any combination of binary mixture where changing the concentration combination synergism altered to inhibition." While the existence of synergism would not be any less interesting, the issue should be acknowledged and addressed.

More minor comments:

1) Are odor mixtures pre-mixed before delivery, or are they mixed in the olfactometer from the individual components? This is a critical detail that should be added to the Methods section.

2) For the linear regression of the PSTH slope, how is it the response duration calculated? Please provide more details.

3) Was the linear regression calculated only for a single representative example (Figures 1D, 2B)? This calculation should be done across samples and presented as a mean +/- standard deviation, perhaps in a table. Were the statistics from Figure 2B from a population, rather than single values from representative traces?

Some details re the figures, references and terminology:

a) Figure 1c, it appears the raw trace and PSTH are not aligned correctly for the Acetone (10^-3) panel (and maybe others?).

b) Figure 2a: alignment issue with the middle (Acetone) column, top two panels.

3) Figure 2a: absolute concentrations should be listed for ease of reading the figure (rather than the blue 'increasing' triangle).

4) For consistency it would be more correct to refer to "Or59b-expressing neurons" and "Or42b-expressing neurons" instead of simply saying responses of Or59b and Or42b.

5) Figure 3C - the authors use a greater than sign (as opposed to less than) when describing the p-values of the various mixtures - please correct throughout the text.

6) A relevant reference that should be cited:

S.A. Ochieng, K.C. Park, T.C. Baker Host plant volatiles synergize responses of sex pheromone-specific olfactory receptor neurons in male Helicoverpa sea PMID 12012103
